# Predictors of human immunodeficiency virus (HIV) infection in primary care among adults living in developed countries: a systematic review

**DOI:** 10.1186/s13643-018-0744-3

**Published:** 2018-06-02

**Authors:** Benhildah N. Rumbwere Dube, Tom P. Marshall, Ronan P. Ryan, Modupe Omonijo

**Affiliations:** 1 Institute of Applied Health Research, University of Birmingham, Birmingham, B15 2TT UK; 2 Public Health England, Health and Wellbeing Directorate, London, UK

**Keywords:** Acquired immuno-deficiency syndrome, Antiretroviral therapies, Diagnosis, Human immunodeficiency virus, Patient characteristics, HIV predictors, Primary care

## Abstract

**Background:**

Early diagnosis of human immunodeficiency virus (HIV) is important because antiretroviral therapies are more effective if infected individuals are diagnosed early. Diagnosis of HIV relies on laboratory testing and determining the demographic and clinical characteristics of undiagnosed HIV-infected patients may be useful in identifying patients for testing. This systematic review aims to identify characteristics of HIV-infected adults prior to diagnosis that could be used in a prediction model for early detection of patients for HIV testing in UK primary care.

**Methods:**

The population of interest was adults aged ≥ 18 years in developed countries. The exposures were demographic, socio-economic or clinical characteristics associated with the outcome, laboratory confirmed HIV/AIDS infection. Observational studies with a comparator group were included in the systematic review. Electronic searches for articles from January 1995 to April 2016 were conducted on online databases of EMBASE, MEDLINE, The Cochrane Library and grey literature. Two reviewers selected studies for inclusion. A checklist was developed for quality assessment, and a data extraction form was created to collate data from selected studies.

**Results:**

Full-text screening of 429 articles identified 17 cohort and case-control studies, from 26,819 retrieved articles. Demographic and socio-economic characteristics associated with HIV infection included age, gender and measures of deprivation. Lifestyle choices identified were drug use, binge-drinking, number of lifetime partners and having a partner with risky behaviour. Eighteen clinical features and comorbid conditions identified in this systematic review are included in the 51 conditions listed in the British HIV Association guidelines. Additional clinical features and comorbid conditions identified but not specified in the guidelines included hyperlipidemia, hypertension, minor trauma and diabetes.

**Conclusion:**

This systematic review consolidates existing scientific evidence on characteristics of HIV-infected individuals that could be used to inform decision making in prognostic model development. Further exploration of availability of some of the demographic and behavioural predictors of HIV, such as ethnicity, number of lifetime partners and partner characteristics, in primary care records will be required to determine whether they can be applied in the prediction model.

**Electronic supplementary material:**

The online version of this article (10.1186/s13643-018-0744-3) contains supplementary material, which is available to authorized users.

## Background

Human immunodeficiency virus (HIV) is a retroviral infection that weakens the immune system and is a subsequent causative agent of acquired immuno-deficiency syndrome (AIDS) [1, 2]. The virus is transmitted through the exchange of a variety of bodily fluids mainly sexually, perinatal and blood-borne [2, 3]. HIV/AIDS is one of the highest contributors to morbidity and the sixth leading cause of mortality worldwide [2, 4]. The World Health Organization (WHO) estimated that 1.5 million people died of HIV/AIDS-related diseases and 36.7 million lived with HIV worldwide, in 2015 [5]. In 2015, it was estimated that 594 deaths were associated with HIV\AIDS in England and 101,200 people were estimated to live with HIV in the UK [6].

The life expectancy of HIV-infected individuals has increased over the years and is approaching that for the general population [7, 8]. This is a result of the effectiveness of antiretroviral therapies (ART) that has led to most individuals coping with HIV infection as a chronic condition rather than an illness inevitably leading to death [9]. The use of ARTs has led to a better quality of life for infected individuals and a reduction in morbidity and mortality [4].

In the 1980s/1990s, more focus was placed on HIV prevention strategies and treatment of symptomatic diseases but due to the benefits of ART, the emphasis has now moved to earlier HIV diagnosis [10]. WHO developed a strategy aimed at reducing new HIV infections, AIDS-related mortality and discrimination to zero with one of the HIV strategies being optimisation of ‘HIV prevention, diagnosis, treatment and care outcomes’ [11].

The CD4 count is an indicator of immunosuppression in an individual infected with HIV [9]. Early diagnosis of people with HIV (cluster of differentiation 4 (CD4) > 350/mm^3^) improves the effectiveness of antiretroviral therapies, and additionally, the treatment and advice provided reduces onward transmission, thereby making late diagnosis of HIV (CD4 < 350/mm^3^) an important public health concern [12, 13]. Furthermore, early diagnosis of HIV and earlier use of therapies reduce health and social care costs by preventing illness associated with HIV [4, 14]. On the other hand, delayed diagnosis of HIV to late stages (CD4 < 350/mm^3^) leads to worse prognosis for the patient due to irreversible immunologic damage and associated problems [13, 15].

Public Health England estimated that out of the 101,200 individuals living with HIV in 2015, 6095 were newly diagnosed and 13% were unaware of their HIV status [6]. In that year, 39% of people that were newly diagnosed with HIV in the UK were detected late (CD4 < 350/mm^3^), which is an intolerably high proportion [6]. Meanwhile, evidence shows that about 33% of patients that are diagnosed with HIV in the UK would have seen a general practitioner (GP) within the previous year [9, 16, 17]. One study found that one in three patients that presented at least one HIV-related symptoms to their GPs was consequently diagnosed with HIV by their GP [18]. Therefore, primary care has a role to play in increasing uptake of HIV diagnostic testing since nearly all the UK population is registered with a GP [19]. HIV testing in general practices can be done by either sending blood samples for laboratory testing or conducting combined HIV antibody and protein 24 (P24) antigen tests followed by laboratory confirmation [9]. However, among those who visit their GP, a challenge is the fact that HIV/AIDS has many signs and symptoms such as rashes, weight loss and respiratory infections and these are not specific to HIV/AIDS.

Current UK guidelines from British HIV Association (BHIVA) recommend HIV testing to individuals from high-risk groups, those with symptoms indicative of HIV or where HIV forms part of the diagnosis [20]. However, approximately three-quarters of patients consult their GPs in the period prior to diagnosis may not present these indicator symptoms and diagnoses [17]. This suggests that these currently recommended predictive factors are of limited use in the identification of possible HIV-infected individuals.

The methods used in routine HIV testing either involve use of screening assays on blood for laboratory testing or rapid tests conducted on samples from a finger-prick or mouth swab at point of care. The commonly used and recommended first-line assays test for HIV antibodies and the HIV p24 antigens simultaneously [9, 20]. These assays can be utilised within a month of HIV infection [9, 20]. The sensitivity of these assay tests ranges from 99.8–100% and the specificity ranges from 99.4–100% [21, 22]. Point-of-care tests (POCTs) are rapid testing devices that diagnose HIV within 15 min. However, such tests have lower specificity in comparison to laboratory tests, thereby giving significantly high proportion of false positives, especially when used in low prevalence settings [9]. It is therefore possible to test for and diagnose HIV using simple blood tests with few false positives and false negatives.

The UK primary care clinicians need to identify patients who should be offered HIV testing. A systematic review is therefore necessary to identify demographic, lifestyle, clinical and laboratory characteristics of patients which might be associated with HIV infection in primary care. The identified characteristics will be investigated to determine if they are documented in electronic primary care records and whether they can be used to predict which primary care patients are likely to have HIV infection.

This systematic review identifies, critically evaluates and interprets available evidence related to the demographic, lifestyle, clinical and laboratory characteristics associated with HIV/AIDS infection in adults in the developed world [23, 24].

## Methods

This systematic review conforms to the requirements of the Preferred Reporting Items for Systematic Reviews and Meta-analyses (Additional file 1, PRISMA) [25]. The methods were detailed in a published protocol, but a summary is included in this section [26]. The PROSPERO registration number for the protocol is CRD42016042427.

### Review question

This systematic review systematically identifies and summarises evidence on characteristics of HIV-infected adults which could be used in a prediction model for early detection of HIV in primary care.

The review question is:

What demographic, lifestyle, clinical and laboratory characteristics are associated with HIV infection in adults aged 18 years and over?

### Population, exposure and outcome

Studies selected included human participants ≥ 18 years. Exposures may be demographic, socio-economic or clinical risk factors or characteristics associated with HIV infection. The comparison group is either people without risk factors or no comparison group. The outcome is laboratory-confirmed HIV/AIDS infection.

### Study design

This review considers observational (analytical) studies, comparing groups and produces predictive values or likelihood ratios (case-control and cohort, both retrospective and prospective studies) [27].

### Search strategy

Studies are identified via electronic searches of EMBASE (Ovid), MEDLINE (Ovid), The Cochrane Library (Wiley) and the unpublished grey literature (SIGLE, Google Scholar and BASE). Additional searches are conducted on abstracts or conference proceedings using Web of Science Conference Proceedings Citation Index (CPCI), Global Index Medicus, guidelines (NICE, DH) and reference searching [28]. There were no language restrictions, and all studies published from year 1995 to April 2016 were included. The search terms used in Ovid MEDLINE (Additional file 2: Appendix I) are adjusted to suit searches in other databases. References were searched and stored using the Refworks referencing programme.

### Inclusion/exclusion criteria

To ensure generalisability to a UK setting, only studies undertaken in the following developed countries are included in this review: Europe (all countries) and North America (USA and Canada), Australia and New Zealand. Studies which include children only are excluded.

### Selection procedure

Two reviewers independently selected articles in the first and second screening of articles. The first screening checked titles/abstracts to find out if articles addressed the review question and fulfilled the inclusion and exclusion criteria (Additional file 3: Appendix II). The second screening was the full article review. Differences between the reviewers were resolved through discussions.

### Quality assessment and data extraction

Quality assessment was done using a checklist for cohort and case-control studies modified from the Scottish Intercollegiate Guidelines Network (SIGN) [29].

A data extraction form was developed to collate data from selected articles. Tabulation and narrative of the results were produced, and the tabulation contains description of the articles (the author, publication year, the study design, number of participants, population under study and outcome).

## Results

### Selection procedure

**Fig. 1:**
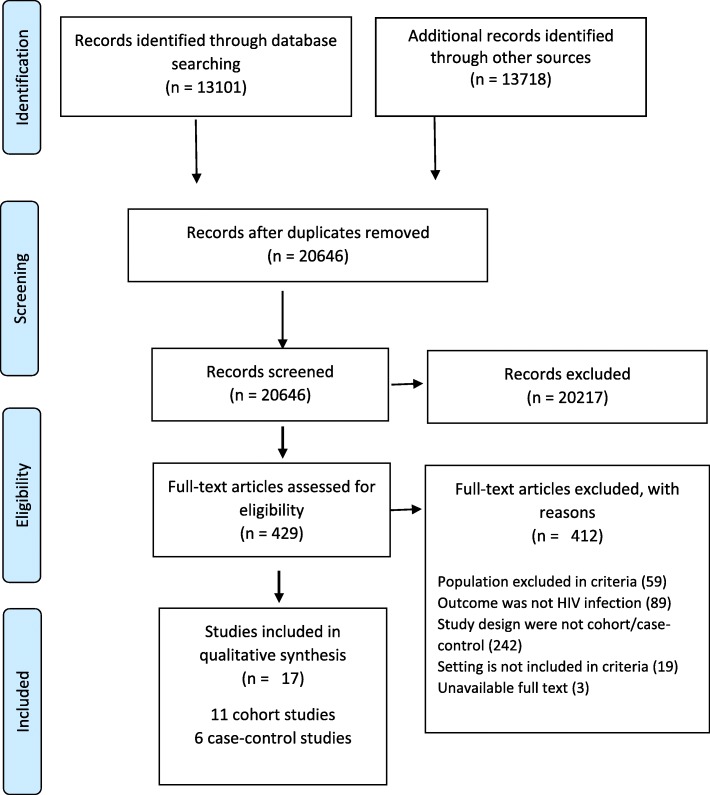
PRISMA 2009 flow diagram

A total of 26,819 hits were returned from the database searches and NICE and DH, 6173 duplicates were removed and 20,646 articles were pre-screened (Fig. 1). The first review resulted in selection of 429 articles using titles/abstracts. A discussion was held to agree on the articles selected. The reviewers independently selected suitable articles using full text and a second discussion was held. The reviewers agreed on 17 articles: 11 cohort and 6 case-control studies.

### Quality of studies

All 11 cohort studies were of acceptable standard, but only 2 were of high quality, in terms of participant recruitment, sample size and how they dealt with bias. The other articles were not clear about how they dealt with confounding factors (Table 1). All 6 case-control studies were of acceptable standard, and half of them were of high quality, in terms of participant recruitment, sample size and how they dealt with bias.

**Table 1:** Data extract and quality assessment summary: cohort and case-control studies

Study	Design	Population, setting	Outcome: duration and follow-up	Study addresses an appropriate and clearly focused question	Participants being studied are selected from the same source populations	Indicate how many people participated	Main potential confounders identified and accounted for	How well was the study done to minimise the risk of bias or confounding?
1. Joore I.K. et al., (2015) [42]	Case-control study	102 cases and 299 controls, Amsterdam, Netherlands	HIV infection: 2002–2012	Yes	Yes	Yes	Cannot say	+
2. Damery S. et al. (2013) [17]	Case-control study	939 cases and 2576 control, UK	HIV/AIDS diagnosis: Jan 1989–Sept 2010	Yes	Yes	Yes	Yes	++
3. Szerlip M.A. et al. (2005) [39]	Case-control study (retrospective)	Older patients aged 55 years and over (53 cases and 106 controls), New Orleans, USA	Diagnosis of HIV infection: 6 months interval up to 12 months	Yes	Yes	Yes	Cannot say	+
4. Ellerbrock T.V. (2004) [30]	Case-control study	217 cases 395 controls, FL, USA	HIV diagnosis: 1998–2000	Cannot say	Yes	Yes	Cannot say	+
5. Burchell, A.N. (2010)	Case-control study	Gay and bisexual men 123 cases and 240 controls, Ontario, Canada	HIV infection: 1998–2006	Yes	Yes	Yes	Yes	++
6. Burchell, A. N. (2003) [41]	Case-control study	Adults aged 18 years and over 80 cases (seroconverts) and 106 controls, Ontario, Canada	Diagnosed HIV infection: June 1998–Dec 2001	Yes	Yes	Yes	Yes	++
7. Hodder, S.L. (2013) [36]	Cohort study (prospective)	*N* = 2099 (women aged 18–44 with 1 or more personal or partner risk factors), USA	HIV prevalence and incidence: 2009–2010 with 6-month follow-up to 12 months	Yes	Yes	Yes	Yes	+
8. Moran. J. (2012) [34]	Cohort study	*N* = 1404 Ireland	HIV infection: 2008–2011	Yes	Cannot say	Yes	No	+
9. Desai M. (2012) [38]	Cohort study	*N* = 328 UK	HIV infection: Sept 2010–Dec 2011	Yes	Cannot say	Yes	No	+
10. Guy R.J. (2011) [35]	Cohort study	*N* = 7857 (MSM) Victoria, Australia	HIV positivity: Apr 2006–Jun 2009	Yes	Yes	Yes	Cannot say	+
11. Krauskopf K. (2011) [45]	Cohort study	*N* = 643 (HIV-infected and at-risk men aged 49 years and older), Bronx, NY, USA	HIV infection: 2001–2006 6-month follow-up	Yes	Yes	Yes	Yes	++
12. Niyonsenga T (2013) [37]	Cohort study	*N* = 20,528 (all cases with HIV/AIDS diagnosis), FL, USA	AIDS/HIV incidence: 1998–2002	Yes	Cannot say	Yes	Cannot say	+
13. Ross, J. D. (1997) [31]	Cohort study	*N* = 8466 (population aged 16 and over), Lothian and Glasgow region of Scotland	HIV positive results: Jan 1989–Dec 1993	Yes		Yes		
14. Gordon S. M. (1995) [32]	Cohort study	*N* = 32 (HIV-positive patients aged ≥ 60) Atlanta, GA, USA	HIV positivity: Jan 1985–July 1992	Yes	No	Yes	No	+
15. Marder K. (1995) [44]	Cohort study (prospective)	Intravenous drug users (99 HIV + ve patients 124 HIV − ve patients), New York City, USA	HIV infection: recruited 1988 and followed up for 3.5 years and 6-month follow-up	Yes	Yes	Yes	Yes	++
16. Hafner J. W. (1997) [33]	Cohort study (retrospective)	*N* = 344 Albuquerque, NM, USA	HIV diagnosis: 19-month period July 1993–Jan 1995	Yes		Yes	Cannot say	+
17. Landau R. (1997) [43]	Cohort study (retrospective)	*N* = 133 (A&E patients aware and unaware of HIV status), London, UK	HIV infection: 1991–1994	Yes	Yes	Yes	No	+

Modified from Scottish Intercollegiate Guidelines Network (SIGN)

Minimise risk of bias or cofounding: high quality (++) □ acceptable (+) □ unacceptable—reject 0

### Study characteristics

The cohort studies were conducted in the UK (3), Ireland (1), Australia (1) and USA (6). The number of participants ranged from 32 to over 20,000 with most studies focusing on patients aged ≥ 18 years. The study duration ranged from 1 to 5 years, but some of the studies did not state follow-up intervals (Table 1).

The case-control studies were conducted in the UK (1), Netherlands (1), the USA (2) and Canada (2). In total, they included 1412 cases and 3423 controls. The study duration ranged from 1 to 12 years with a 6-month follow-up for most of the studies.

### Identified predictors of HIV infection

The predictors of HIV identified were categorised into demographic and socio-economic, behavioural or lifestyle, clinical features and comorbidities. Statistically significant characteristics or those with highest percentages were included.

#### Demography and socio-economic

The significant demographic characteristics (Table 2) associated with HIV infection were (i) homosexuals and/or bisexuals, mainly men who have sex with men (MSM) (5 studies) 1.8 to 2.7 times risk [30–34], (ii) black ethnicity (1 study); 6.8 times risk [30] and (iii) age ranges (3 studies), mainly between 27 and 40 years with up to 11.5 times the risk [31, 35, 36]. Two studies revealed that gender had no significant association with the risk of HIV infection [30, 31]. Two studies showed conflicting evidence on the increased risk of HIV infection associated with country of birth; one study from the USA showed that being born in the USA was associated with 1.76 times the risk [30], but a study from Australia showed that being born in Australia had a non-significant risk [35].

**Table 2:** Demographic characteristics identified in selected studies

Studies		Ellerbrock 2004 [30]	Guy 2011 [35]	Hodder 2013 [36]	Ross 1997 [31]	Niyonsenga 2013 [37]	Gordon 1995 [32]	Desai 2012 [38]	Hafner 1997 [33]	Moran 2012 [34]
		OR	OR	OR	OR	CC**	%		%	%
Demographic										
Age	Reference group		< 30 y	18–26 years	21–25 years					
	26–30				1.7 (1.05–2.8)					
	27–33			5.83 (1.22–27.96)						
	30–39		1.91 (1.27-2.87)							
	31–35				0.3*					
	34+			11.54 (2.71–49.05)						
	36–40				1.6*					
	40+		1.81 (1.19-2.75)							
Ethnicity	Black race (Reference = white)	6.77 (4.17–11)								
	Aboriginal or Torres Strait Islander		1.68* (0.41–6.94)							
Country of birth	Born in USA	1.76 (1.22–2.53)								
	Born in Australia		1.42* (1.00–2.02)							
Sexuality	Homosexual/bisexual	1.79* (0.67–4.79)			2.7 (1.5–4.8)		37%		57%	61%
	Heterosexual	1.00			1.0				3%	28%
Socio-economic factor										
Housing problems								17%		
Poverty index in rural areas						− 0.25*				
Poverty index in urban areas						0.58				
Annual income < $10,000		13.2 (7.91–22)								
Farmworker		2.09 (1.47–2.96)								
Unemployed		5.08 (3.18–8.14)						26%		
Education beyond high school				0.43* (0.15–1.24)						
Not a high school graduate		2.15 (1.48–3.1)								

*NB* % do not add up to 100% because they are provided for all risk factors

*Not statistically significant

**Correlation coefficient

Socio-economic conditions associated with increased risk of HIV identified were (i) poverty in urban but not in rural areas (1 study) [37], (ii) annual income under $10,000 having 13 times the risk (1 study) [30], (iii) unemployment (1 study) [30], (iv) housing problems (1 study) [30] and (v) not being a high school graduate or having low education attainment (2 studies); 2.2 times the risk [30, 38].

#### Behavioural characteristics

Behavioural characteristics (Table 3) associated with an increased risk of HIV infection can be categorised into personal lifestyle, partner lifestyle and effects of life events. Personal lifestyle choices identified were (i) injecting drugs (7 studies); 2 to 21 times the risk [30, 31] [32–36], (ii) smoking crack cocaine (1 study); 22.8 times the risk [30], (iii) being a current smoker (1 study) [38], (iv) binge-drinking (1 study); 12.8 times the risk [34], (v) exchanging money or drugs for sex (1 study); 19 times the risk [30], (vi) male anal sex (1 study); 1.6 times the risk [35] and (vii) being obese (1 study) [30]. Personal sexual behaviours identified were unsafe sex (2 studies); 1.8 times the risk [35, 38] and having multiple sex partners (1 study); 5.5 times the risk for males with ≥ 10 and 20 times the risk for females with ≥ 3 lifetime partners [30]. Partner-related behaviours identified were (i) HIV-positive partner (2 studies); 3 and 8 times the risk [35, 36], (ii) partner’s use of illicit drugs (2 studies); 1.57 and 17 times [30, 36], (iii) partner’s alcohol dependence/binge-drinking (1 study); 1.4 to 1.8 times the risk [39].

**Table 3:** Behavioural or lifestyle––personal choices identified in selected studies

Predictor	Ellerbrock 2004 [30]	Gordon 1995 [32]	Guy 2011 [35]	Hafner 1997 [33]	Hodder 2013 [36]	Moran 2012 [34]	Ross 1997 [31]	Desai 2012 [38]	Szerlip 2005 [39]
	OR	%	OR	%	OR	%	OR	%	OR
Injected drugs users	21.1 (4.89–90.9)	18%	2.97 (1.77–5.00)	30%	2.71 (1.33–5.53)	10%	2.3 (1.5–3.5)		
Ever smoked crack cocaine	22.8 (12.6–41.5)								
Binge-drinking or alcohol misuse					1.57* (0.74–3.33)				12.8 (1.65–99)
Substance use (combined)**					2.52 (1.22–5.21)			22%	
Current smokers								25%	
Unsafe sex			1.84 (1.6– 3.20)					60%	
HIV positive partner			3.24 (1.47–7.11)						
Sex with drug user	17.2 (7.18–40.9)								
Contact abroad							2*		
Ever exchanged money or drugs for sex	19.3 (11.2–33.2)								
Male anal sex in the last ≥ 6 months			1.63 (1.13– 2.35)						
Multiple life partners	M: 5.51 (3.18–9.55) F: 19.8 (8.81–44.2)								
Obesity								10%	

*Not statistically significant

**Includes drug use or binge-drinking

One study revealed risk-associated stressful events in men having sex with men to be; (i) the number of stressful events, (ii) events occur in ages under 30 years associated with 7 times the risk, (iii) type of stressful events such as bereavement and death of close friend and financial crisis and relationship breakdown (romantic and other relations); 3 times the risk [40].

#### Clinical features

Evidence from 4 studies (Table 4) revealed that HIV infection was associated with clinical features: (i) flu-like symptoms including fever/chills and cough (3 studies); 4.5 times the risk [33, 39, 41], (ii) rash (1 study); 4.5 times the risk [39], (iii) weight loss (2 studies); 13 to 39 times the risk [17, 41], (iv) diarrhoea (2 studies); 2 to 4.4 times the risk [17, 41] and one study identified abdominal pain, minor trauma and nausea/vomiting as the condition affecting 5–6% of the HIV-positive patients [33].

**Table 4:** Clinical features and comorbidities identified in selected studies

Condition			Damery 2013 [17]	Joore 2015 [42]	Hafner 1997 [33]	Marder 1995 [44]	Burchell 2003 [41]	Szerlip 2005 [39]	Krauskopf 2011 [45]	Landau 1997 [43]	Hodder 2013 [36]	Guy 2011 [35]	Ellerbrock 2004 [30]
			OR (CI)	OR (CI)	%	OR	% & OR	OR	%	%	OR	OR	OR
Clinical features													
Weight loss			13.4 (5.15–6.7)	39.6 (6.2–∞)									
Fever or chills				4.5* (0.5–54.3)	13%								
Cough					7%								
Flu-like symptoms							76%						
Diarrhoea				2* (0.2–17.4)									
Diarrhoea consultation	one only		3.7* (0.9–5.48)										
Diarrhoea consultation	two		4.4 (2.3–2.81)										
Abdominal pain					5%								
Minor trauma					6%								
Nausea/vomiting					6%								
Rash							4.5						
Number of HIV indicator conditions	One			11.7 (6–23.6)									
	Two			77.5 (18.2–700.8)									
Comorbidities													
Respiratory	Pneumonia		47.7 (3.54–52)	8.3 (2–49.8)									
	Pneumocystis carinii									52%			
Dermatology	Psoriasis			2.9* (0.1–∞)									
	Psoriasis—one consultation only		2.6 (1.69–1.5)										
	Psoriasis—two consultations		3 (1.38–2.5)										
	Herpes zoster		25.4 (5.76–14.2)	10.9 (2–108.9)									
Neurology	Peripheral neuropathy			15.9 (2–∞)									
	Neurologic disability in women					2.4							
	Neurologic disability in men					1.9 (1.1–3.2)							
Gastroenterology	Oral candidiasis		29.4 (4.57–21.8)	7.1* (0.6–∞)									
	Hepatitis B			11.5 (1.2–∞)				8.3 (2.65–26.2)					
	Chronic liver disease								22% (15%–29%)				
Oncology	Non-Hodgkin’s lymphoma		12.6 (2.13–15)										
	Lymphogranuloma venereum			7.1* (0.6–∞)									
Gynaecology	Cervical dysplasia			2.9* (0.4–232.4)									
	Condyloma acuminata			12.1 (1.2–600.9)									
Haematology	Leucocytopenia			11.5 (1.2–∞)									
	Blood dyscrasia		5.7 (2.44–4)										
ENT	Lymphadenopathy		11.3 (5.15–5.3)	29.8 (4.4–∞)									
	Parotitis		8.6 (1.68–11)										
Other	Mononucleosis-like illness			6.2 (1.6–29)									
	Pyrexia of unknown origin		7.2 (4.05–3.5)										
	Hyperlipidemia								25% (17%–32%)				
	Hypertension								10% (4%–16%)				
	Diabetes								10% (5%–14%)				
	Sexually Transmitted Infection (STI)		10.8 (3.38–7.6)					10.1 (3.39–30.12)					10.1 (6.89–14.9)
	STI diagnosis	≤ 2 years										2.72 (1.77–4.2)	
		≤ 14 days										3.19 (2.05–4.96)	
	Number of STIs per patient	One		14.6 (5.5–45.6)									
		≥ 2		37.9 (5.6-∞)									
	Syphilis			39.3 (5.7–1703.9)				7.35 (2.52–21.5)					12.7 (7.28–22.3)
	Seropositive for syphilis*												7.29 (4.15–12.8)
	Infectious Syphilis diagnosis	≤ 2 years										3.86 (1.99–7.5)	
		≤ 14 days										4.9 (2.51–9.56)	
	Chlamydia			11.8 (3–67.5)									
	Chlamydia diagnosis	≤ 2 years										2.31 (1.4–3.81)	
		≤ 14 days										2.62 (1.56–4.39)	
	Gonorrhoea			15.9 (2–∞)									6.51 (4.4–9.65)
	Genital herpes			2.9* (0.1–∞)									

*Not statistically significant

∞ Means infinity upper limit

#### Comorbidities associated with HIV

The clinical indicator conditions (Table 4) were categorised into the following: respiratory, dermatology, neurology, gastroenterology, gynaecology, haematology, ophthalmology, ear, nose and throat (ENT) and other (not classified).

The respiratory conditions identified were pneumonia (2 studies); 8 and 48 times the risk [17, 41] and pneumocystis in 52% of the HIV-infected patients (1 study) [42]. The dermatological conditions identified were psoriasis (2 studies); 2.6 to 3 times the risk [17, 41] and herpes zoster (2 studies); 10.9 and 25.4 times the risk [17, 41].

The evidence revealed that HIV infection was significantly associated with peripheral neuropathy (1 study); 15.9 times the risk [41] and neurologic disabilities cranial nerve abnormalities and fine limb movement (1 study); 2.4 times the risk in women and 1.9 times the risk in men [43]. The gastroenterological conditions identified were oral candidiasis (2 studies); 7.1 and 29.4 times the risk [17, 41], hepatitis B (2 studies); 8.3 and 11.5 times the risk [44, 41] and liver diseases (1 study), affecting 22% of the HIV-infected patients [45]. One oncological conditions identified was Non-Hodgkin’s lymphoma (1 study); 12.6 times the risk [17].

Only one study identified gynaecological conditions associated with increased risk of HIV diagnosis and condyloma acuminata; 12.1 times the risk [41]. The two haematological conditions identified in the studies were leukocytopenia (1 study); 11.5 times the risk [41] and blood dyscrasia (1 study); 5.7 times the risk [17]. ENT conditions identified were lymphadenopathy (2 studies); 11.3 and 29.8 times the risk [17, 41] and parotitis (1 study); 8.6 times the risk [17].

The other conditions identified were mononucleosis-like illness (1 study); 6.2 times the risk [41], pyrexia of unknown origin (1 study); 7.2 times the risk [17] and one study which had 10–25% of the HIV-infected patients with hyperlipidemia, hypertension and diabetes [45]. The other conditions identified were sexually transmitted infections (5 studies), 2.7 to 37.9 times the risk [17, 30, 35, 44, 41], and the following infections were identified: (i) syphilis (3 studies), 3.9 to 39.3 times the risk [30, 44, 41]; (ii) chlamydia (2 studies), 2.3 to 11.8 times the risk [30, 35]; (iii) gonorrhoea (2 studies), 6.5 to 15.9 times the risk [30, 41] and (iv) genital herpes (1 study), 2.9 times the risk [41].

## Discussion

This systematic review identified 10 demographic and socio-economic characteristics, 11 behavioural characteristics, and 27 clinical features and comorbid conditions that are significantly associated with HIV infection.

The purpose of this systematic review was to identify predictors of HIV infection available in electronic patient records that could be incorporated in a prediction model to identify primary care patients with undiagnosed HIV. Candidate predictors identified are either routinely recorded in electronic primary care records or require further investigation to assess if they can be reliably identified and included in a future clinical prediction model (Table 5).

**Table 5:** Predictors identified and availability in electronic primary care records

Category of predictor	Predictor of HIV infection	Likelihood of being recorded in primary care records
Sociodemographic	Age	Present for all patients
	Gender	Present for all patients
	Social status	Inferred from postcode
	Poverty index	Present as deprivation quintile
	Annual income	Inferred from prescription payments, benefits
	Employment status	Likely to be poorly recorded
	Sexual orientation	Require further investigation
	Not a high school graduate	Not present
	Country of birth	Not present
	Ethnicity	Present for some patients
Behavioural	Smoking status	Very likely to be present
	Drug use	Present for some patients
	Binge-drinking or alcohol misuse	Present for some patients
	Obesity	Very likely to be present
	Contact abroad	Might be present
	Stressful events	Present for some patients
	Unsafe sex	Likely not present
	Ever exchanged money or drugs for sex	Likely not present
	Male anal sex	Likely not present
	Number of lifetime partners	Likely not present
	Partner characteristics	Likely not present
Clinical and comorbid conditions	Weight loss	Likely to be present
	Fever or chills	Likely to be present
	Cough	Likely to be present
	Flu like symptoms	Likely to be present
	Diarrhoea	Likely to be present
	Abdominal pain	Likely to be present
	Minor trauma	Likely to be present
	Nausea/vomiting	Likely to be present
	Rash	Likely to be present
	Pneumonia	Likely to be present
	Pneumocystis carinii	Likely to be present
	Psoriasis	Likely to be present
	Herpes zoster	Likely to be present
	Peripheral neuropathy	Likely to be present
	Neurologic disability	Likely to be present
	Oral candidiasis	Likely to be present
	Hepatitis B	Likely to be present
	Chronic liver disease	Likely to be present
	Non-Hodgkin’s lymphoma	Likely to be present
	Condyloma acuminata	Likely to be present
	Leucocytopenia	Likely to be present
	Blood dyscrasia	Likely to be present
	Lymphadenopathy	Likely to be present
	Parotitis	Likely to be present
	Mononucleosis-like illness	Likely to be present
	Pyrexia of unknown origin	Likely to be present
	Hyperlipidemia	Likely to be present
	Hypertension	Likely to be present
	Diabetes	Likely to be present
	Sexually transmitted infection	Likely to be present
	Syphilis	Likely to be present
	Chlamydia	Likely to be present
	Gonorrhoea	Likely to be present
	Genital herpes	Likely to be present

The demographic and socio-economic predictors identified and available in primary care records are age, gender and deprivation quintile as a proxy for some of the socio-economic predictors. Behavioural predictors identified and available in electronic health records are drug use, binge-drinking or alcohol misuse, current smokers and obesity. All the clinical features and comorbid diseases identified are most probably available in electronic health records (Table 5).

Some of the demographic, socio-economic and behavioural predictors identified in literature, such as ethnicity, country of birth, income and education levels, might be available in primary care records and therefore require further investigation on completeness.

### Limitations

This systematic review focused on studies conducted in developed countries whereas most of the studies on HIV predictors were conducted in developing countries, mostly in Africa. Most of the studies conducted on HIV were case studies, qualitative studies and cross-sectional studies which are not suitable in identifying risk factors.

Some of the studies identified in this systematic review reported percentages rather than odds ratio in their results making the interpretation of risk association difficult.

## Conclusion

This systematic review revealed existing scientific evidence on predictors of HIV that can be used to inform decision making in prognostic model development [46]. Only 2 demographic and socio-economic characteristics (age and gender) and 4 behavioural characteristics (drugs use, binge-drinking or alcohol misuse, current smokers and obesity) identified in literature are available in electronic primary-care records. The other 8 demographic and socio-economic and 7 behavioural characteristics require further investigation on completeness or if they are not available at all. Further exploration will determine whether the characteristics can be applied in a model.

Of the 51 clinical conditions in BHIVA guidelines, 18 were identified as significant predictors of HIV infection in this systematic review. The following predictors identified in literature are not included in the guidelines: fever/chills/flu-like symptoms, cough, abdominal pain, minor trauma, nausea/vomiting, rash, hyperlipidemia, hypertension and diabetes.

## Additional files

Additional file 1: PRISMA 2009 Checklist. (DOC 63 kb)

Additional file 2: Appendix I. Search strategy. (DOCX 12 kb)

Additional file 3: Appendix II. Selection criteria. (DOCX 14 kb)

## Abbreviations

AIDS: Acquired immuno-deficiency syndrome
ART: Antiretroviral therapies
CD4 count: Cluster of differentiation 4 count
ENT: Ear, nose and throat
GP: General practitioner
HIV: Human immunodeficiency virus
MSM: Men who have sex with men
NAAT: Nucleic Acid Amplification Test
P24 antigen tests: Protein 24 antigen tests
PCR: Polymerase chain reaction
POCTs: Point-of-care tests
PRISMA: Systematic Reviews and Meta-analyses
PRISMA-P 2015: Systematic Reviews and Meta-analyses for Protocols 2015
SIGN: Scottish Intercollegiate Guidelines Network
STI: Sexually transmitted infection
USA: Unites States of America

## References

[1] Sharp PM, Hahn BH. Origins of HIV and the AIDS pandemic. Cold Spring Harb Perspect Med. 2011;1(1):a006841. 10.1101/cshperspect.a006841. 10.1101/cshperspect.a006841 PMC3234451 22229120

[2] World Health Organisation (WHO). HIV/AIDS factsheet. Geneva: World Health Organisation; 2015. Available: http://www.who.int/en/news-room/fact-sheets/detail/hiv-aids. Accessed 31 Oct 2015.

[3] Hessol NA, Gandhi M, Greenblatt RM (2005). Epidemiology and natural history of HIV infection in women, U.S. Department of Health and Human Services, Health Resources and Services Administration, HIV/AIDS Bureau.

[4] European Centre for Disease Prevention and Control (ECDC). HIV testing: increasing uptake and effectiveness in the European Union. Stockholm: ECDC; 2010.

[5] World Health Organisation (WHO) (2016). Global Health Observatory (GHO) data: HIV/AIDS. [online].

[6] Kirwan PD, Chau C, Brown AE, Gill ON (2016). Delpech VC and contributors. HIV in the UK––2016 report.

[7] Nakagawa F, May M, Phillips A. Life expectancy living with HIV: recent estimates and future implications. Curr Opin Infect Dis. 2013;26(1):17–25. 10.1097/QCO.0b013e32835ba6b1 23221765

[8] Simmons RD, Ciancio BC, Kall MM, Rice BD, Delpech VC (2013). Ten-year mortality trends among persons diagnosed with HIV infection in England and Wales in the era of antiretroviral therapy: AIDS remains a silent killer. HIV Medicine.

[9] Madge S, Matthews P, Singh S, Theobald N (2011). HIV in primary care.

[10] Girardi E, Sabin CA, Monforte DD (2007). Late diagnosis of HIV infection: epidemiological features, consequences and strategies to encourage earlier testing. J Acquir Immune Defic Syndr.

[11] World Health Organisation (WHO). Global update on the health sector response to HIV. Geneva, Switzerland, 2014. Available: http://www.who.int/hiv/pub/progressreports/update2014/en/ [Accessed on 15 Nov 2016].

[12] Fernandez M, Collazo JB, Bowen GS, Varga LM, Hernandez N, Perrino T. Predictors of HIV testing and intention to test among Hispanic Farmworkers in South Florida. J Rural Health. 2005;21(1):56–64. 10.1111/j.1748-0361.2005.tb00062.x 15667010

[13] Department of Health (DH) (2013). A framework for sexual health improvement in England.

[14] Halve it. Early testing saves lives: HIV is a public health priority. 3rd edition. Gilead Sciences Ltd, n.d. Available: http://www.bhiva.org/documents/Publications/Halve_it_Position_Paper.pdf. Accessed 31 Oct 2015.

[15] Sanders GD, Bayoumi AM, Sundaram V, Bilir SP, Neukermans CP, Rydzak CE, Douglass LR, Lazzeroni LC, Holodniy M, Owens DK (2015). Cost-effectiveness of screening for HIV in the era of highly active antiretroviral therapy. N Engl J Med.

[16] Sudarshi D, Pao D, Murphy G (2008). Missed opportunities for diagnosing primary HIV infection. Sex Transm Infect.

[17] Damery S, Nichols L, Holder R, Ryan R, Wilson S, Warmington S, Stokes-Lampard H, Manavi K. Assessing the predictive value of HIV indicator conditions in general practice: a case–control study using the THIN database. Br J Gen Pract. 2013;63(611):370–7. 10.3399/bjgp13X668159 PMC3662453 23735407

[18] Wellesley R, Whittle A, Figueroa J, Anderson J, Castles R, Boomla K, Griffiths C, Leber W. Does general practice deliver safe primary care to people living with HIV? A case-notes review. Br J Gen Pract. 2015;65(639):e655–61. 10.3399/bjgp15X686905 PMC4582878 26412842

[19] Burns FM, Johnson AM, Nazroo J (2008). Missed opportunities for earlier HIV diagnosis within primary and secondary healthcare settings in the UK. AIDS.

[20] British HIV Association (BHIVA) (2008). UK national guidelines for HIV testing.

[21] Ly TD, Ebel A, Faucher V, Fihman V, Laperche S (2007). Could the new HIV combined p24 antigen and antibody assays replace p24 antigen specific assays?. J Virol Methods.

[22] Fanmi AN, Ramière C, Tardy JC, André P (2013). Real-life evaluation of a human immunodeficiency virus screening algorithm using a single combined p24 antigen–antibody assay. Eur J Clin Microbiol Infect Dis.

[23] Norman I, Griffiths P (2014). The rise and rise of the systematic review. Int J Nurs Stud.

[24] Trivedi D, Goodman C, Dickinson A, Gage H, McLaughlin J, Manthorpe J, Ashaye K, Iliffe S (2013). A protocol for a systematic review of research on managing behavioural and psychological symptoms in dementia for community-dwelling older people: evidence mapping and syntheses. Syst Rev.

[25] Moher D, Liberati A, Tetzlaff J, Altman DG (2009). Preferred reporting items for systematic reviews and meta-analyses: the PRISMA statement. BMJ.

[26] Rumbwere Dube BN, Marshall TM, Ryan RP (2016). Predictors of human immunodeficiency virus (HIV) infection in primary care: a systematic review protocol. Syst Rev.

[27] Glasziou P, Irwig L, Bain C, Colditz G (2001). Systematic reviews in health care: a practical guide.

[28] Bruce N, Pope D, Stanistreet D (2008). Quantitative research methods for health research: a practical interactive guide to epidemiology and statistics.

[29] Scottish Intercollegiate Guidelines Network. Critical appraisal notes and checklists. [Online] Available: http://www.sign.ac.uk/checklists-and-notes.html. Accessed 14 Jan 2016.

[30] Ellerbrock TV, Chamblee S, Bush TJ, Johnson JW, Marsh BJ, Lowell P, Trenschel RJ, Von Reyn CF, Johnson LS, Horsburgh CR (2004). Human immunodeficiency virus infection in a rural community. Am J Epidemiol.

[31] Ross JD, Goldberg DJ (1997). Patterns of HIV testing in Scotland: a general practitioner perspective. Scott Med J.

[32] Gordon SM, Thompson S (1995). The changing epidemiology of human-immunodeficiency-virus infection in older persons. J Am Geriatr Soc.

[33] Hafner JW, Brillman JC (1997). Symptomatology of HIV-related illness and community-acquired illness in an HIV-infected emergency department population. Ann Emerg Med.

[34] Moran J, Connell J, Burke D, infection HWEHIV (2012). Demographic features and risk factors among individuals with early HIV infection in Ireland 2008-2011. Ir J Med Sci.

[35] Guy RJ, Spelman T, Stoove M, ElHayek C, Goller J, Fairley CK, Leslie D, Tee BK, Roth N, Grulich AE, Hellard ME (2011). Risk factors for HIV seroconversion in men who have sex with men in Victoria, Australia: results from a sentinel surveillance system. Sex Health.

[36] Hodder SL, Justman J, Hughes JP, Wang J, Haley DF, Adimora AA, Del Rio C, Golin CE, Kuo I, Rompalo A, Soto-Torres L, Mannheimer SB, Johnson-Lewis LT, Eshleman SH, El-Sadr WM, Womens HIV (2013). SeroIncidence study HIV acquisition among women from selected areas of the United States: a cohort study. Ann Intern Med.

[37] Niyonsenga T, Trepka MJ, Lieb S, Maddox LM (2013). Measuring socioeconomic inequality in the incidence of AIDS: rural-urban considerations. AIDS Behav.

[38] Desai M, Bevin MA, Holland H, Darling D, Mukela A, Menon Johansson A, Fox J (2012). Understanding the health needs and risk behaviours of new HIV patients. HIV Med.

[39] Burchell AN, Calzavara LM, Myers T, Remis RS, Raboud J, Corey P and Swantee C, "Stress and increased HIV infection risk among gay and bisexual men," AIDS, vol. 24, p. 1757–1764, 2010. 10.1097/QAD.0b013e32833af7c9 20543662

[40] Burchell AN, Calzavara LM, Myers T, Remis RS, Raboud J, Corey P, Swantee C. Stress and increased HIV infection risk among gay and bisexual men. AIDS. 2010;24(11):1757–64. 10.1097/QAD.0b013e32833af7c9 20543662

[41] Burchell AN, Calzavara L, Ramuscak N, Myers T, Major C, Rachlis A, Gough K, Raboud J, Remis RS, Polaris HIV Seroconversion Study Team (2003). Symptomatic primary HIV infection or risk experiences? Circumstances surrounding HIV testing and diagnosis among recent seroconverters. Int J STD AIDS.

[42] Joore IK, Arts DL, Kruijer MJP, Van Charante EPM, Geerlings SE, Prins JM, Van Bergen JE (2015). HIV indicator condition-guided testing to reduce the number of undiagnosed patients and prevent late presentation in a high-prevalence area: a case–control study in primary care. Sex Transm Infect.

[43] Landau R, Coker R, Vermeulen E, Touquet R, Fothergill J, Poznansky MC (1997). Patients unaware of their HIV status present to an inner city accident and emergency department with respiratory complications. Accid Emerg Med.

[44] Marder K, Liu XH, Stern Y, Malouf R, Dooneief G, Bell K, Todak G, Joseph M, Sorrell S, Sadr WE, Williams JBW, Ehrhardt A, Stein Z, Gorman J (1995). Risk of human-immunodeficiency-virus type 1-related neurologic disease in a cohort of intravenous-drug-users. Arch Neurol.

[45] Krauskopf K, Federman DA, Mhango G, Klein RS (2011). Association of HIV status and non-AIDS comorbid diagnoses in a cohort of older HIV-infected and at-risk men. J Gen Intern Med.

[46] Wright RW, Brand RA, Dunn W, Spindler KP (2007). How to write a systematic review. Clin Orthop Relat Res.

