# Supplementary material for: Predictors of human immunodeficiency virus (HIV) infection in primary care among adults living in developed countries: a systematic review
Source: Syst Rev. 2018 Jun 2;7:82. doi: 10.1186/s13643-018-0744-3 (PMC5985063; doi:10.1186/s13643-018-0744-3)
Supplement: Supplementary file 2 — Appendix I. Search strategy. (DOCX 12 kb) [file 13643_2018_744_MOESM2_ESM.docx]

## **Appendix I: Search strategy**

The following search strategy used in Ovid MEDLINE will be used and in some cases adjusted to suit searches in different databases;

1 Human Immunodeficiency Virus.mp.

2 HIV/

3 Acquired immuno Deficiency Syndrome.mp.

4 AIDS.mp.

5 exp Acquired Immunodeficiency Syndrome/

6 *HIV/

7 aids.mp. or *Acquired Immunodeficiency Syndrome/

8 *Acquired Immunodeficiency Syndrome/

9 sign.mp.

10 signs.mp.

11 Symptom Flare Up/ or symptom.mp.

12 symptoms.mp.

13 risk factor$.mp. or Risk Factors/

14 clinical indicat$.mp.

15 clinical feature$.mp.

16 predict$.mp.

17 risk score$.mp.

18 model.mp.

19 1 or 2 or 3 or 4 or 5 or 6 or 7 or 8

20 9 or 10 or 11 or 12 or 13 or 14 or 15 or 16 or 17 or 18

21 primary care.mp. or exp Primary Health Care/

22 Rural Health/ or Nurse Practitioners/ or Family Practice/ or family practic$.mp. or Diagnosis/ or Physicians/

23 Physician-Patient Relations/ or Family Practice/ or Physician's Role/ or general practic$.mp.

24 21 or 22 or 23

25 19 and 20 and 24

26 limit 25 to humans
