# Supplementary material for: Predictors of human immunodeficiency virus (HIV) infection in primary care among adults living in developed countries: a systematic review
Source: Syst Rev. 2018 Jun 2;7:82. doi: 10.1186/s13643-018-0744-3 (PMC5985063; doi:10.1186/s13643-018-0744-3)
Supplement: Supplementary file 3 — Appendix II. Selection criteria. (DOCX 14 kb) [file 13643_2018_744_MOESM3_ESM.docx]

## **Appendix II: Screening criteria**

## **Screening stage 1 criteria**

Screen using the title/abstracts and find out if studies fit the eligibility criteria

**Eligibility criteria**

1. The exposure of the study are risk factors or characteristics associated with HIV infection

- demographic,
- socio-economic or
- clinical

1. The outcome of the study is

- Human Immunodeficiency Virus or HIV or Acquired Immuno-deficiency Syndrome or AIDS

1. Published 1995 onwards
2. Reviews, cohort studies, case-control, RCTs

**Exclusion criteria**

1. Non-human studies.
2. Children (under 18) only
3. Non-HIV diagnosis
4. Setting is developing countries only

## **Screening stage 2 criteria**

Screen using the whole article and find out if studies fit the eligibility criteria

**Eligibility criteria**

1. The exposure of the study are risk factors or characteristics associated with HIV infection

- demographic,
- behavioural
- socio-economic or
- clinical

1. The outcome of the study is

- Human Immunodeficiency Virus or HIV or Acquired Immuno-deficiency Syndrome or AIDS

1. Published 1995 onwards
2. Reviews, cohort studies, case-control, RCTs

**Exclusion criteria**

1. Non-human studies.
2. Children (under 18) only
3. Non-HIV diagnosis or post HIV diagnosis
4. Setting is developing countries only
5. Studies on Health care or treatment
